# Robot-assisted retroperitoneal lymph node dissection as primary treatment for stage II seminoma germ cell tumor

**DOI:** 10.1590/S1677-5538.IBJU.2023.0389

**Published:** 2024-03-18

**Authors:** Stefano Cogo Badan, Willy Baccaglini, Arie Carneiro, Gustavo Caserta Lemos

**Affiliations:** 1 Albert Einstein Instituto Israelita de Ensino e Pesquisa Departamento de Urologia São Paulo SP Brasil Departamento de Urologia, Albert Einstein Instituto Israelita de Ensino e Pesquisa, São Paulo, SP, Brasil;; 2 Faculdade de Medicina do ABC Disciplina de Urologia Santo André SP Brasil Disciplina de Urologia, Faculdade de Medicina do ABC - FMABC, Santo André, SP, Brasil;; 3 Hospital Israelita Albert Einstein São Paulo SP Brasil Serviço de Urologia, Hospital Israelita Albert Einstein, , São Paulo, SP, Brasil

## Abstract

**Introduction:**

Historically, therapeutic avenues for patients with clinical stage II seminoma germ cell tumors (SGCT) were confined to radiotherapy and chemotherapy. While survival rates with these modalities are commendable, both entail substantial long-term morbidities. Furthermore, this youthful patient cohort exhibits elevated rates of secondary malignancies, surfacing decades post-successful primary cancer treatment (1). Recently, retroperitoneal lymph node dissection (RPLND) has emerged as a primary treatment consideration for individuals with low-volume metastatic seminoma (2–4). However, there is a dearth of video documentation illustrating the robotic assisted (RA) bilateral approach (5- 7).

**Methods:**

We present the case of a 24-year-old male who underwent prior left orchiectomy for seminoma (pT1b). Despite negative serum tumor markers, a 1.7 x 1.4cm lymph node enlargement was identified in the aortic bifurcation after 4 months, classifying the patient as stage IIA per the IGCCCG risk classification. Subsequently, a RA bilateral template RPLND was performed due to the patient's refusal of chemotherapy, citing concerns about offspring.

**Results:**

The surgery was performed, incorporating nerve sparing techniques, lasting 4h13minutes, an estimated bleeding rate of 400ml, without intraoperative complications. The patient was discharged within 24 hours of the procedure, following a prescribed low-fat diet.

**Conclusion:**

The patient experienced postoperative well-being, painlessness, and resumed work three weeks post-procedure. Preserved ejaculation was noted, and adjuvant therapy was performed with 2 cycles of EP due to the anatomopathological result. The feasibility of robotic primary RPLND for SGCT was demonstrated, showing reduced postoperative pain and early hospital discharge. Further studies are necessary to validate our findings regarding oncological, safety, and functional outcomes.

Available at: http://www.intbrazjurol.com.br/video-section/20230389_Badan_et_al

**Int Braz J Urol. 2024; 50 (Video #2): 225-6**

## References

[1] Alsyouf M, Daneshmand S (2022). Clinical stage II seminoma: management options. World J Urol.

[2] Heidenreich A, Paffenholz P, Hartmann F, Seelemeyer F, Pfister D (2024). Retroperitoneal Lymph Node Dissection in Clinical Stage IIA/B Metastatic Seminoma: Results of the COlogne Trial of Retroperitoneal Lymphadenectomy In Metastatic Seminoma (COTRIMS). Eur Urol Oncol.

[3] Daneshmand S, Cary C, Masterson T, Einhorn L, Adra N, Boorjian SA (2023). Surgery in Early Metastatic Seminoma: A Phase II Trial of Retroperitoneal Lymph Node Dissection for Testicular Seminoma With Limited Retroperitoneal Lymphadenopathy. J Clin Oncol.

[4] Hiester A, Che Y, Lusch A, Kuß O, Niegisch G, Lorch A (2023). Phase 2 Single-arm Trial of Primary Retroperitoneal Lymph Node Dissection in Patients with Seminomatous Testicular Germ Cell Tumors with Clinical Stage IIA/B (PRIMETEST). Eur Urol.

[5] Heidenreich A, Paffenholz P, Nestler T, Pfister D, Daneshmand S (2020). Role of primary retroperitoneal lymph node dissection in stage I and low-volume metastatic germ cell tumors. Curr Opin Urol.

[6] Hiester A, Nini A, Arsov C, Buddensieck C, Albers P (2020). Robotic Assisted Retroperitoneal Lymph Node Dissection for Small Volume Metastatic Testicular Cancer. J Urol.

[7] Santos VE, Fornazieri L, Brazão ES, Pinto PR, da Costa WH, Zequi SC (2023). Primary laparoscopic RPLND for pure seminona metastasis: feasibility of supine and lateral approaches. Int Braz J Urol.

